# 2-Hydroxylation of *Acinetobacter baumannii* Lipid A Contributes to Virulence

**DOI:** 10.1128/IAI.00066-19

**Published:** 2019-03-25

**Authors:** Toby L. Bartholomew, Timothy J. Kidd, Joana Sá Pessoa, Raquel Conde Álvarez, José A. Bengoechea

**Affiliations:** a Wellcome-Wolfson Institute for Experimental Medicine, Queen’s University Belfast, Belfast, United Kingdom; b School of Chemistry and Molecular Biosciences, The University of Queensland, Brisbane, Australia; c Child Health Research Center, The University of Queensland, Brisbane, Australia; d Instituto de Salud Tropical y Departamento de Microbiología y Parasitología, Facultad de Medicina, Universidad de Navarra, Edificio de Investigación, Universidad de Navarra, c/Irunlarrea, Pamplona, Spain; University of California, Davis

**Keywords:** *Acinetobacter*, lipid A, virulence

## Abstract

Acinetobacter baumannii causes a wide range of nosocomial infections. This pathogen is considered a threat to human health due to the increasingly frequent isolation of multidrug-resistant strains. There is a major gap in knowledge on the infection biology of A. baumannii, and only a few virulence factors have been characterized, including lipopolysaccharide. The lipid A expressed by A. baumannii is hepta-acylated and contains 2-hydroxylaurate. The late acyltransferases controlling the acylation of lipid A have been already characterized. Here, we report the characterization of A. baumannii LpxO, which encodes the enzyme responsible for the 2-hydroxylation of lipid A. By genetic methods and mass spectrometry, we demonstrate that LpxO catalyzes the 2-hydroxylation of the laurate transferred by A. baumannii LpxL. LpxO-dependent lipid A 2-hydroxylation protects A. baumannii from polymyxin B, colistin, and human β-defensin 3. LpxO contributes to the survival of A. baumannii in human whole blood and is required for pathogen survival in the waxmoth Galleria mellonella. LpxO also protects *Acinetobacter* from G. mellonella antimicrobial peptides and limits their expression. Further demonstrating the importance of LpxO-dependent modification in immune evasion, 2-hydroxylation of lipid A limits the activation of the mitogen-activated protein kinase Jun N-terminal protein kinase to attenuate inflammatory responses. In addition, LpxO-controlled lipid A modification mediates the production of the anti-inflammatory cytokine interleukin-10 (IL-10) via the activation of the transcriptional factor CREB. IL-10 in turn limits the production of inflammatory cytokines following A. baumannii infection. Altogether, our studies suggest that LpxO is a candidate for the development of anti-A. baumannii drugs.

## Abstract (precis)

Acinetobacter baumannii causes a wide range of nosocomial infections. This pathogen is considered a threat to human health due to the increasingly frequent isolation of multidrug-resistant strains.

## INTRODUCTION

Lipopolysaccharide (LPS), located in the outer leaflet of the outer membrane (OM), is composed of three regions: the lipid A moiety, the core oligosaccharide, and the O-polysaccharide. Hexa-acylated lipid A, such as the canonical lipid A structure expressed by Escherichia coli K-12 (1), is recognized by the Toll-like receptor 4 (TLR4)-MD2 complex, resulting in the activation of downstream signaling cascades leading to cytokine production and subsequent inflammation (2). The lipid A structure can be modified by pathogens in response to different conditions (1, 3). Well-characterized modifications are the addition or removal of fatty acids, alteration of the phosphate groups, or additions of other chemical groups, such as amino acids and sugars (1, 3). These modifications may result in resistance to cationic antimicrobial peptides (CAMPs), reduced or increased activation of inflammatory responses, or greater protection against disturbances in pH or desiccation (2, 3).

Acinetobacter baumannii is an opportunistic Gram-negative pathogen causing a wide range of nosocomial infections, although the most common clinical manifestations comprise ventilator-associated pneumonia and central line-associated bloodstream infections (4). A. baumannii is considered a global threat to human health due to the increasing isolation of multidrug-, extensively drug-, and even pan-drug-resistant strains. Colistin and tigecycline have become the last-line treatment for multidrug-resistant A. baumannii (5). Despite its clinical relevance, there is a major knowledge gap on the infection biology of A. baumannii, and only a few virulence factors have been characterized, including LPS (6). A. baumannii synthesizes lipooligosaccharide (LOS), rather than LPS, due to the absence of the O-polysaccharide. Structurally, A. baumannii lipid A consists of a β(1′-6)-linked disaccharide of glucosamine phosphorylated at the 1 and 4′ positions with positions 2, 3, 2′, and 3′ acylated with R-3-hydroxymyristoyl groups. The 2 and 3′ R-3-hydroxymyristoyl groups are further acylated with laurate (C_12_) through the action of the late acyltransferase LpxM, whereas the 2’ R-3-hydroxymyristoyl group is acylated with laurate (C_12_) through the action of the late acyltransferase LpxL. Thus, in contrast to many Gram-negative pathogens, lipid A of A. baumannii is predominantly hepta-acylated (7–11). The evidence demonstrates that hepta-acylation of A. baumannii lipid A promotes resistance to CAMPs, contributes to desiccation survival, and is important for A. baumannii virulence (7). This lipid A is recognized by the TLR4/MD2 receptor complex and triggers the activation of NF-κB and mitogen-activated protein kinases (MAPKs) to induce inflammation and antibacterial molecules such as defensins (7, 8).

A. baumannii can modify its lipid A with phosphoethanolamine and galactosamine, and these modifications are implicated in colistin resistance (9–11). PmrC/EptA is responsible for the addition of phosphoethanolamine to lipid A (9), whereas NaxD is required for the modification with galactosamine (10). The PmrAB two-component system governs expression of PmrC and NaxD, and there are reports of clinical isolates harboring mutations in PmrA or PmrB leading to the upregulation of the system with a concomitant increase in the replacement of the lipid A with phosphoethanolamine and galactosamine (9–11). Colistin resistance is also mediated through complete loss of the LOS by loss-of-function mutations within genes essential for lipid A biosynthesis (*lpxA, lpxC*, or *lpxD*) (12). However, LOS-deficient strains of A. baumannii show a dramatic reduction in virulence, and not all strains possess the ability to mutate early-stage lipid A biosynthetic enzymes (13).

All of the published studies on A. baumannii lipid A report the presence of 2-hydroxylaurate (7–11). Few Gram-negative bacteria have been shown to produce lipid A species containing hydroxylated secondary acyl chains, and the evidence suggests that this lipid A modification enables the pathogens to successfully infect their hosts. In Vibrio cholerae, the late acyltransferase LpxN is responsible for transferring the hydroxylated laurate to the lipid A domain (14), whereas in Bordetella bronchiseptica, Salmonella enterica serovar Typhimuirum, and Klebsiella pneumoniae, the incorporation of a hydroxyl group in a fatty acid is catalyzed by the enzyme LpxO (15–17). Interestingly, *in silico* analysis of A. baumannii genomes revealed the presence of an *lpxO* homolog, suggesting that A. baumannii LpxO is responsible for the lipid A modification with 2-hydroxylaurate. In this work, we characterized A. baumannii *lpxO*, which is required for the hydroxylation of lipid A. This study demonstrates that LpxO plays an important role in A. baumannii infection biology, since LpxO-dependent lipid A modification protects the pathogen from CAMPs, mediates resistance to phagocytosis, and limits the activation of host defense responses in invertebrates and mammalian cells.

## RESULTS

### A. baumannii LpxO 2-hydroxylates lipid A.

**FIG 1:**
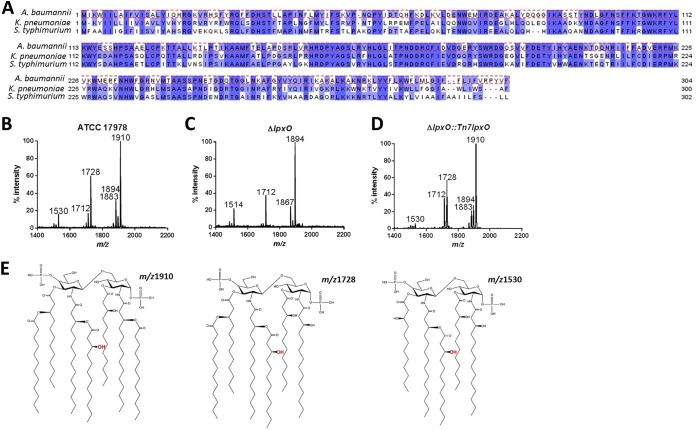
A. baumannii LpxO 2-hydroxylates lipid A. (A) A. baumannii encodes an LpxO homolog with 62% and 59% sequence similarity to K. pneumoniae and S. enterica serovar Typhimurium LpxO, respectively. Shown are negative-ion MALDI-TOF mass spectrometry spectra of lipid A purified from A. baumannii ATCC 17978 (B), A. baumannii Δ*lpxO* mutant (Δ*lpxO*) (C), and A. baumannii Δ*lpxO*::Tn*7lpxO* mutant (Δ*lpxO*::Tn*7lpxO*) (D). Data represent the mass-to-charge ratios (*m/z*) of each lipid A species detected and are representative of three extractions. (E) Proposed lipid A structures of the species produced by wild-type A. baumannii based on previous publications (7–11).

*In silico* analyses of A. baumannii genomes revealed that this pathogen carries one homolog of K. pneumoniae and S. enterica serovar Typhimurium *lpxO*. Analysis of the genome of A. baumannii strain ATCC 17978 showed that LpxO (locus tag A1S_0308) is 62 and 59% identical to K. pneumoniae and Salmonella enterica serovar Typhimurium LpxO, respectively (Fig. 1A). To confirm that the identified loci are indeed responsible for the 2-hydroxylation of lipid A, A. baumannii strain ATCC 17978 *lpxO* was mutated by double recombination. Control experiments showed that the wild type and *lpxO* mutant had similar growth kinetics in both rich and minimal media (see Fig. S1 in the supplemental material). Lipid A was extracted from the wild type and the *lpxO* mutant using an ammonium hydroxide-isobutyric acid method and subjected to negative-ion matrix-assisted laser desorption ionization–time of flight (MALDI-TOF) mass spectrometry. Lipid A produced by the wild type contained the species with mass-to-charge ratios (*m/z*) of 1,910, 1,728, and 1,530 (Fig. 1B). These molecular species have been previously found in lipid A of A. baumannii strains (7–11), and the proposed chemical structures are shown in Fig. 1E. In contrast, the *lpxO* mutant produced a lipid A which contained the species *m/z* 1,894, 1,712, and 1,514, an *m/z* difference of 16 compared to the wild-type species (i.e., *m/z* 1,910, 1,728, and 1,530, respectively), which corresponded with the lack of ions containing 2-hydroxylaurate (C_12:OH_) (Fig. 1C). Complementation of the mutant restored the production of wild-type lipid A (Fig. 1D), demonstrating that *lpxO* is responsible for the 2-hydroxylation of A. baumannii lipid A.

We sought to determine whether other membrane lipids were affected in the membrane of the *lpxO* mutant. However, the analysis of the lipid composition by thin-layer chromatography did not reveal any differences in the levels of cardiolipin, phosphatidylglycerol, or phosphoethanolamine between the membranes of the wild type and *lpxO* mutant (Fig. S2A).

To assess whether the changes in the lipid A structure observed in the *lpxO* mutant affect the outer membrane permeability to hydrophobic agents, we measured the partition of the hydrophobic fluorescent probe (1-*N*-phenylnaphthylamine; NPN) into the cell membrane. NPN is excluded by the intact bacterial OM but exhibits increased fluorescence after partitioning into disrupted OMs (18). Thus, an increase in fluorescence indicates alterations in the OM. However, we did not observe any differences in the uptake of NPN between the wild type and the *lpxO* mutant (Fig. S2B), suggesting that no major functional alterations occur in the OM of the *lpxO* mutant.

### A. baumannii LpxO hydroxylates the laurate linked to the 2’ R-3-hydroxymyristoyl group of lipid A.

**FIG 2:**
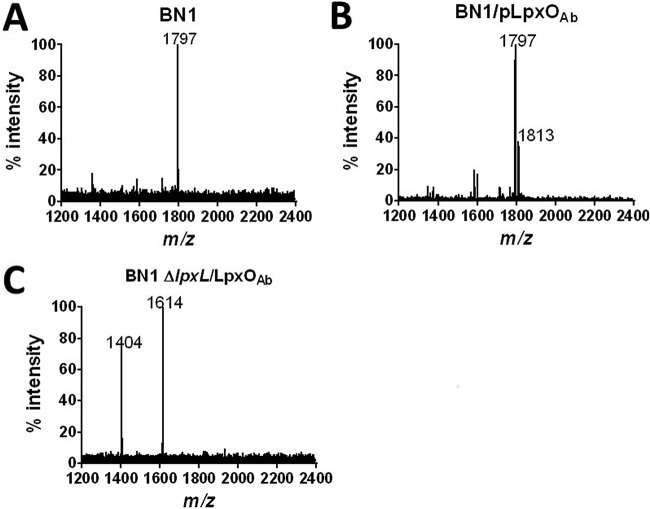
A. baumannii LpxO modifies the LpxL-transferred lauryl group. Shown are negative-ion MALDI-TOF mass spectrometry spectra of lipid A purified from E. coli BN1 (A), E. coli BN1::Tn*7lpxO* mutant (BN1::Tn7*lpxO*) (B), and E. coli BN1-Δ*lpxL*::Tn*7lpxO* mutant (BN1- Δ*lpxL*::Tn*7lpxO*) (C). Data represent the *m/z* of each lipid A species detected and are representative of three extractions.

Recently, Boll and coworkers (7) have shown that 2-hydroxylation in A. baumannii lipid A is absent from an *lpxL_Ab_* mutant, suggesting that LpxO hydroxylates the 2’-*R*-hydroxylaurate group. To validate this hypothesis, we used a genetic approach by expressing LpxO in E. coli BN1 (19). This E. coli strain produces the canonical hexa-acylated lipid A of *m/z* 1,797 (Fig. 2A), corresponding to a β(1′-6)-linked disaccharide of glucosamine phosphorylated at the 1 and 4′ positions, with positions 2, 3, 2′, and 3′ being acylated with R-3-hydroxymyristoyl groups. The 2′ and 3′ R-3-hydroxymyristoyl groups are further acylated with laurate (C_12_) and myristate (C_14_) (19). Expression of A. baumannii LpxO in E. coli BN1 resulted in the hydroxylation of the hexa-acylated lipid A species to give *m/z* 1,813 (Fig. 2B). In contrast, LpxO-mediated 2-hydroxylation was not observed when LpxO was expressed in the E. coli BN1 *lpxL* mutant (Fig. 2C), confirming that LpxO hydroxylates the laurate fatty acid chain transferred by LpxL.

### LpxO-dependent lipid A modification increases A. baumannii resistance to antimicrobial peptides.

**FIG 3:**
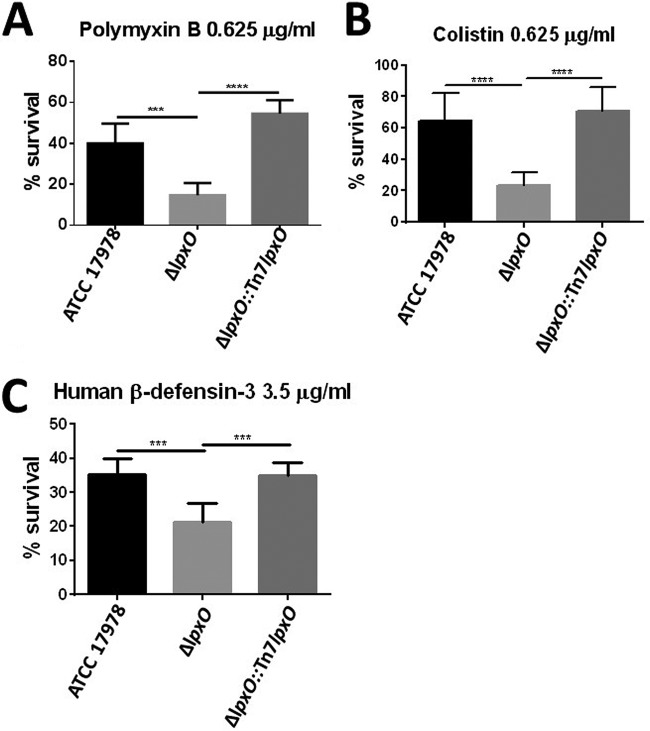
Deletion of *lpxO* decreases A. baumannii resistance to CAMPs. Shown is percent survival of A. baumannii ATCC 17978, A. baumannii Δ*lpxO* mutant (Δ*lpxO*), and A. baumannii Δ*lpxO*::Tn*7lpxO* mutant (Δ*lpxO*::Tn7*lpxO*) following 1 h of exposure to polymyxin B (A), colistin (B), and human β-defensin 3 (C). Values are presented as the means ± standard deviations (SD) from three independent experiments measured in duplicate. *P* values were <0.01 (***) and <0.001 (****) for the indicated comparisons using one-way ANOVA with Bonferroni contrasts; n.s., not significant.

Polymyxins B and E (also known as colistin) interact with LPS by electrostatic interactions, causing a disruption of the OM barrier (20, 21). Polymyxin B is most often used as a topical ointment, whereas colistin is becoming the antibiotic of last resort to treat multidrug-resistant infections, including those triggered by A. baumannii. We sought to determine whether LpxO-dependent lipid A modification protects A. baumannii from polymyxins. Results displayed in Fig. 3 demonstrate that the *lpxO* mutant was more susceptible than the wild type to both polymyxin B and colistin. Complementation restored wild-type levels of resistance, suggesting that LpxO-dependent hydroxylation contributes to A. baumannii resistance to polymyxins.

Human antimicrobial peptides such as defensins are weapons of the innate immune system against infections. They do share with polymyxins the initial interaction with the LPS (20), and we therefore hypothesized that LpxO-mediated lipid A modification increases resistance to human antimicrobial peptides. To test this hypothesis, we exposed A. baumannii strains to β-defensin 3 and determined the survival after 1 h of incubation. Human β-defensin 3 is active against multidrug-resistant bacteria, and it has been reported to increase severalfold in the lungs upon infection (22–24). We observed increased killing of the *lpxO* mutant by β-defensin 3 (Fig. 3C). The enhanced killing was abrogated in the complemented strain, indicating that LpxO-mediated lipid A modification is associated with resistance to β-defensin 3.

Altogether, our findings demonstrate that LpxO-governed 2-hydroxylation of A. baumannii lipid A promotes resistance to human antimicrobial peptides and clinically relevant polymyxins.

### LpxO contributes to survival of A. baumannii in human whole blood.

**FIG 4:**
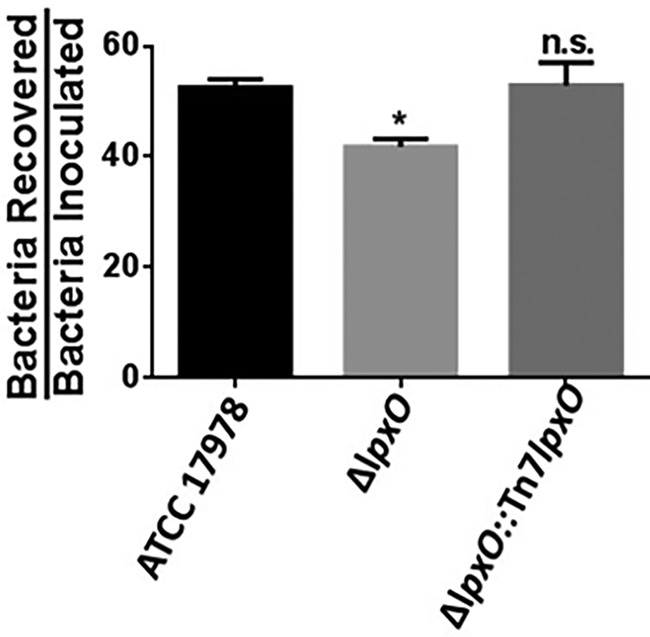
Deletion of *lpxO* increases human phagocyte-mediated killing of A. baumannii. Three hundred μl of fresh human blood (from three different donors) was mixed with 1 × 10^7^ CFU of A. baumannii ATCC 17978, A. baumannii Δ*lpxO* mutant (Δ*lpxO*), and A. baumannii Δ*lpxO*::Tn*7lpxO* mutant (Δ*lpxO*::Tn*7lpxO*) and incubated at 37°C for 3 h. The bacterial counts recovered were then divided by the initial counts. Experiments were performed with duplicate samples on three independent occasions. *, *P* < 0.05 versus A. baumannii ATCC 17978, determined using one-way ANOVA with Bonferroni contrasts; n.s., not significant.

Bacterial killing assays in whole blood allow the *ex vivo* assessment of the interaction between pathogens and professional phagocytes. We then sought to determine whether LpxO contributes to A. baumannii survival in whole human blood. The *lpxO* mutant was recovered in significantly lower numbers than the wild type (Fig. 4), whereas there were not differences between the complemented and wild-type strains. These results demonstrate that the decreased survival of the *lpxO* mutant in whole human blood is associated with the lack of 2-hydroxylation in lipid A.

### A. baumannii *lpxO* is attenuated in the Galleria mellonella infection model.

**FIG 5:**
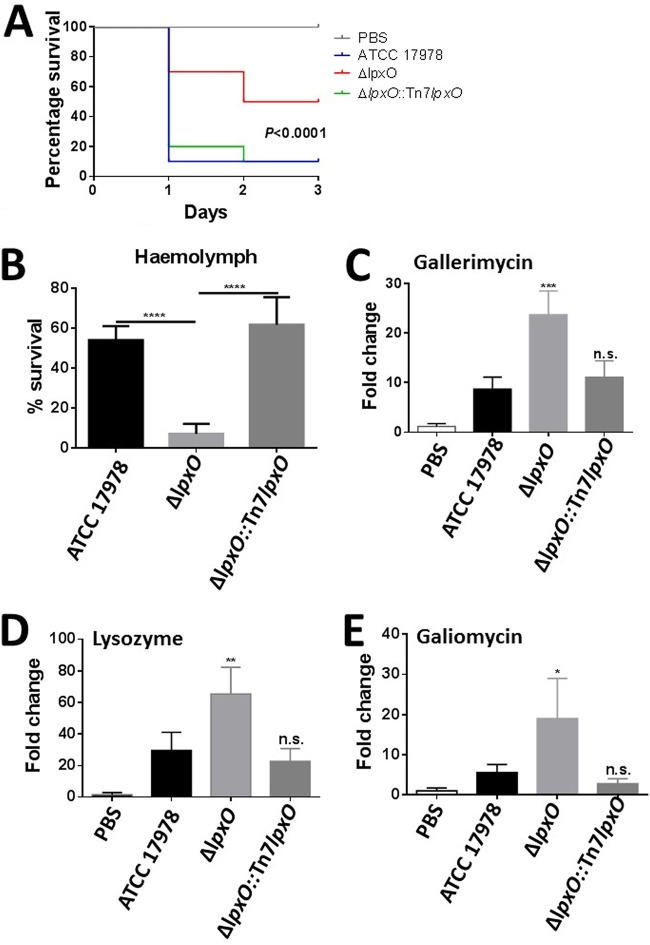
A. baumannii *lpxO* mutant displays decreased virulence in the G. mellonella waxworm infection model. (A) Percent survival of G. mellonella over 72 h postinfection with 5 × 10^4^ organisms of A. baumannii ATCC 17978, A. baumannii Δ*lpxO* mutant (Δ*lpxO*), and A. baumannii Δ*lpxO*::Tn*7lpxO* mutant (Δ*lpxO*::Tn*7lpxO*). Thirty larvae were infected in each group. Level of significance was determined using the log-rank (Mantel-Cox) test with Bonferroni correction for multiple comparisons. (B) Percent survival of A. baumannii ATCC 17978, A. baumannii Δ*lpxO* mutant (Δ*lpxO*), and A. baumannii Δ*lpxO*::Tn*7lpxO* mutant (Δ*lpxO*::Tn*7lpxO*) following 1 h of exposure to G. mellonella hemolymph obtained from larvae challenged with heat-killed E. coli. (C to E) G. mellonella antimicrobial peptide expression determined after 12 h of infection with A. baumannii ATCC 17978, A. baumannii Δ*lpxO* mutant (Δ*lpxO*), and A. baumannii Δ*lpxO*::Tn*7lpxO* mutant (Δ*lpxO*::Tn*7lpxO*) by reverse transcriptase quantitative real‐time PCR. Three larvae per group were infected, and values are presented as the means ± SD from two independent cDNA preparations measured in duplicate. In panels B to E, *P* values were <0.05 (*),  0.01 (**), <0.001 (***), and <0.0001 (****) versus A. baumannii ATCC 17978, determined using one-way ANOVA with Bonferroni contrasts.

The G. mellonella infection model is widely used to assess the virulence of A. baumannii (25). Moreover, there is good correlation between virulence in G. mellonella and that in the mouse model (26, 27). Equal numbers of CFU of all three strains were injected into G. mellonella, and survival of the larvae was monitored over several days. Inoculation with sterile PBS into the larvae resulted in no mortality (Fig. 5). Three days postinoculation, only 10% of the *Galleria* organisms survived when challenged with the wild-type and complemented strains; in contrast, 50% of the larvae survived when inoculated with the *lpxO* mutant (Fig. 5).

CAMPs are part of the G. mellonella defenses activated upon infection (28). A few hours after infection, multiple CAMPs are synthesized and released into the hemolymph to neutralize bacterial infection (28–30). Therefore, we sought to determine whether the *lpxO* mutant is susceptible to these antimicrobial peptides. G. mellonella was challenged with heat‐killed Escherichia coli to boost the expression of antimicrobial peptides (29, 30), and after 24 h, the hemolymph rich in CAMPs was collected and used to assess the susceptibility of the A. baumannii strains by a broth microdilution assay. Results shown in Fig. 5B demonstrate that the *lpxO* mutant was more susceptible to G. mellonella peptides than the wild type. Complementation restored the wild-type levels of resistance to the hemolymph peptides, indicating that the 2-hydroxylation of A. baumannii lipid A promotes resistance to a repertoire of CAMPs produced in response to infections.

We have previously demonstrated that there is a correlation between virulence of Gram-negative pathogens and the expression of G. mellonella antimicrobial peptides (29, 30). The reduced virulence of the *lpxO* mutant led us to assess the expression of CAMPs in G. mellonella larvae infected with the *lpxO* mutant. After 12 h of infection, the expression levels of gallerimycin, galiomycin, and lysozyme were significantly higher in larvae infected with the *lpxO* mutant than in larvae infected with the wild-type strain (Fig. 5C to E). Complementation restored the expression of the peptides to wild-type levels, suggesting that the 2-hydroxylation of A. baumannii lipid A is associated with an attenuated expression of G. mellonella CAMPs.

Altogether, our findings provide evidence that LpxO-mediated 2-hydroxylation of A. baumannii lipid A is necessary for the virulence of this pathogen in G. mellonella by limiting the production of CAMPs and promoting resistance to them.

### Hydroxylated A. baumannii lipid A reduces inflammatory responses in macrophages.

**FIG 6:**
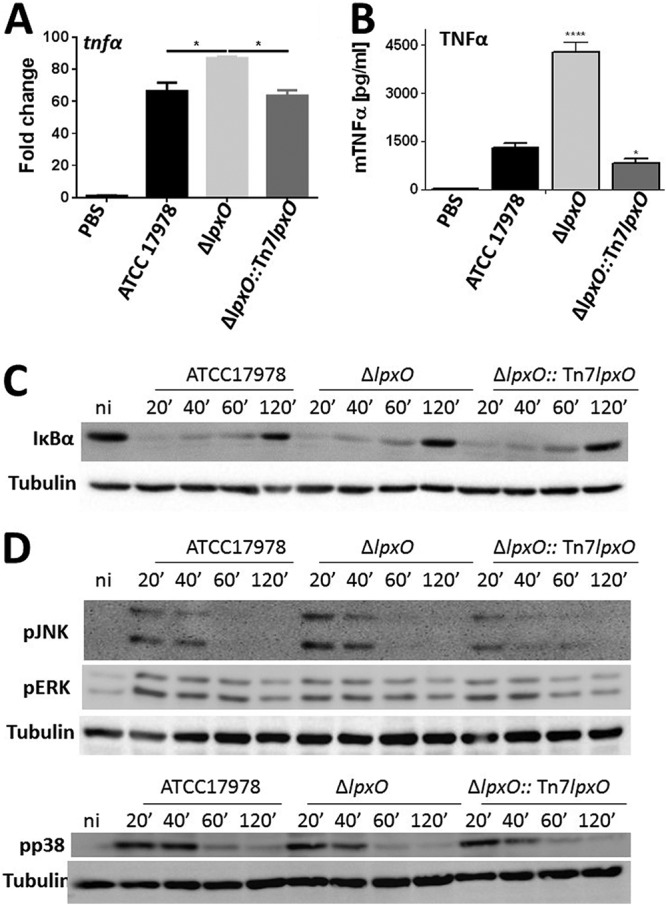
*lpxO* deletion in A. baumannii results in upregulation of inflammatory responses in macrophages upon infection. (A) *tnf*α expression in iBMDMs infected for 5 h with UV-killed A. baumannii ATCC 17978, A. baumannii Δ*lpxO* mutant (Δ*lpxO*), and A. baumannii Δ*lpxO*::Tn*7lpxO* mutant (Δ*lpxO*::Tn*7lpxO*) by reverse transcriptase quantitative real‐time PCR. Values are presented as the means ± SD from three independent cDNA preparations measured in duplicate. (B) TNF-α secretion by iBMDMs stimulated for 5 h with UV-killed A. baumannii ATCC 17978, A. baumannii Δ*lpxO* mutant (Δ*lpxO*), and A. baumannii Δ*lpxO*::Tn*7lpxO* mutant (Δ*lpxO*::Tn*7lpxO*). In panels A and B, *P* values were <0.05 (*) and <0.0001 (****) versus A. baumannii ATCC 17978, determined using one-way ANOVA with Bonferroni contrasts. (C) Immunoblot analysis of IκBα and tubulin levels in lysates of iBMDMs infected with UV-killed A. baumannii ATCC 17978, A. baumannii Δ*lpxO* mutant (Δ*lpxO*), and A. baumannii Δ*lpxO*::Tn*7lpxO* mutant (Δ*lpxO*::Tn*7lpxO*) for the indicated times. (D) Immunoblot analysis of phospho‐JNK (pJNK), phospho‐ERK (pERK), phosphor-p38 (pp38), and tubulin levels in lysates of iBMDMs infected with UV-killed A. baumannii ATCC 17978, A. baumannii Δ*lpxO* mutant (Δ*lpxO*), and A. baumannii Δ*lpxO*::Tn*7lpxO* mutant (Δ*lpxO*::Tn*7lpxO*). In panels C and D, data are representative of at least three independent experiments.

The lipid A pattern is recognized by the TLR4/MD2 complex, leading to the activation of innate signaling pathways and resulting in inflammation and clearance of the infection. However, many pathogens alter the LPS molecular pattern to evade detection by the TLR4/MD2 complex to reduce inflammation (2). Therefore, we sought to investigate whether LpxO-dependent lipid A modification helps A. baumannii to limit the activation of inflammatory responses. Figure 6A shows that the expression of *tnf*α was higher in immortalized bone marrow-derived macrophages (iBMDMs) infected with the *lpxO* mutant than in iBMDMs infected with the wild type. As anticipated, the *lpxO* mutant induced higher levels of tumor necrosis factor alpha (TNF-α) than the wild type (Fig. 6B). Complementation restored the expression and production of cytokines to wild-type levels, indicating that the heightened inflammation induced by the *lpxO* mutant is due to the lack of 2-hydroxylation in lipid A.

The activation of NF-κB and MAPK governed inflammatory responses following infection. We then sought to determine the signaling pathways activated by the *lpxO* mutant. iBMDMs were infected with the wild type, the *lpxO* mutant, and the complemented strain, with the activation of NF‐κB and MAPKs assessed by immunoblotting. In the canonical NF‐κB activation pathway, nuclear translocation of NF‐κB is preceded by phosphorylation and subsequent degradation of IκBα. All strains triggered the degradation of IκBα (Fig. 6C), and no differences were observed between strains. Whereas the three strains triggered the phosphorylation of the MAPKs p38, extracellular signal-regulated kinase (ERK), and Jun N-terminal protein kinase (JNK) (Fig. 6B), the phosphorylation of JNK was increased in macrophages infected with the *lpxO* mutant. Image analysis showed a 22 and 281% increase in the phosphorylation of JNK 20 and 40 min postinfection with the *lpxO* mutant, respectively, compared to the phosphorylation induced by the wild type. The complemented strain induced phosphorylation of JNK similar to that of the wild type at all time points. Control experiments showed that A. baumannii induction of TNF-α is dependent on the activation of JNK, because the levels of this cytokine were significantly lower in the supernatants of infected macrophages treated with the JNK inhibitor SP600125 (Fig. S3). Collectively, these results indicate that the 2-hydroxylation of lipid A limits the activation of MAPK JNK to attenuate inflammatory responses.

**FIG 7:**
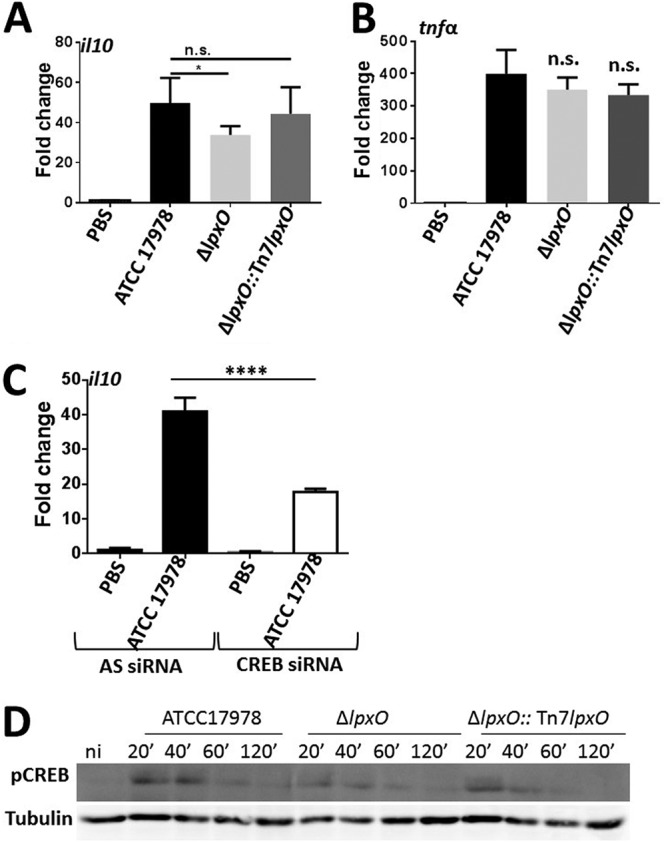
*lpxO* deletion in A. baumannii results in a decrease in *il10* expression. (A) *il10* expression in iBMDMs infected for 5 h with UV-killed A. baumannii ATCC 17978, A. baumannii Δ*lpxO* mutant (Δ*lpxO*), and A. baumannii Δ*lpxO*::Tn7*lpxO* mutant (Δ*lpxO*::Tn7*lpxO*) by reverse transcriptase quantitative real‐time PCR. Values are presented as the means ± SD from three independent cDNA preparations measured in duplicate. (B) *tnf*α expression in *il10^−/−^* iBMDMs infected for 5 h with UV-killed A. baumannii ATCC 17978, A. baumannii Δ*lpxO* mutant (Δ*lpxO*), and A. baumannii Δ*lpxO*::Tn7*lpxO* mutant (Δ*lpxO*::Tn7*lpxO*) by reverse transcriptase quantitative real‐time PCR. Values are presented as the means ± SD from two independent cDNA preparations measured in duplicate. (C) *il10* expression in control (AS) and CREB siRNA-transfected iBMDMs infected for 5 h with UV-killed A. baumannii ATCC 17978 by reverse transcriptase quantitative real‐time PCR. Values are presented as the means ± SD from two independent cDNA preparations measured in duplicate. (D) Immunoblot analysis of phosphor-CREB (pCREB) and tubulin levels in lysates of iBMDMs infected with UV-killed A. baumannii ATCC 17978, A. baumannii Δ*lpxO* mutant (Δ*lpxO*), and A. baumannii Δ*lpxO*::Tn7*lpxO* mutant (Δ*lpxO*::Tn7*lpxO*). Data are representative of at least three independent experiments. In panels A to C, *P* values were <0.05 (*) and <0.0001 (****) versus A. baumannii ATCC 17978, determined using one-way ANOVA with Bonferroni contrasts. n.s., not significant.

Interestingly, the expression of the anti-inflammatory cytokine *il10* was significantly reduced in iBMDMs infected with the *lpxO* mutant, whereas we did not observe any difference in *il10* levels triggered by the wild-type and complemented strains (Fig. 7A). These results suggested that the 2-hydroxylation of A. baumannii lipid A contributes to the induction of the anti-inflammatory cytokine interleukin-10 (IL-10). To investigate whether the reduced levels of IL-10 triggered by the *lpxO* mutant could be responsible for the increased inflammatory response activated by this mutant, we infected *il10^−/−^* iBMDMs and assessed inflammatory responses. No differences were observed in the levels of inflammatory cytokines induced by the wild type and *lpxO* mutant (Fig. 7B), supporting the notion that the increased inflammation triggered by the *lpxO* mutant is due to a decrease in the levels of the anti-inflammatory cytokine IL-10.

We next sought to determine the signaling pathway governing the production of IL-10 in A. baumannii-infected macrophages. The transcriptional factor CREB controls the expression of IL-10 (31), and MAPK p38 and ERK have been shown to cooperate in IL-10 induction following infection (32). Since we have already shown that A. baumanii induced the activation of p38 and ERK (Fig. 6D), we focused on investigating the potential role of CREB to control IL-10 production in A. baumanii-infected cells. Suppression of endogenous expression of CREB using CREB-specific short interfering RNA (siRNA) led to a significant decrease in the levels of *il10* in macrophages infected with the wild-type strain (Fig. 7C), indicating that CREB indeed does govern A. baumannii induction of IL-10. Remarkably, immunoblot analysis revealed that the phosphorylation of CREB was decreased in macrophages infected with the *lpxO* mutant, in sharp contrast to that of cells infected with the wild type (Fig. 7D). Image analysis showed a 19 and 29% decrease in the phosphorylation of CREB 20 and 40 min postinfection with the *lpxO* mutant compared to that triggered by the wild type. Complementation of the *lpxO* mutant restored the phosphorylation of CREB to wild-type levels (Fig. 7D).

Altogether, these findings demonstrate that the 2-hydroxylation of A. baumannii lipid A mediates the production of the anti-inflammatory cytokine IL-10 via the activation of CREB. IL-10, in turn, limits the production of inflammatory cytokines following A. baumannii infection.

## DISCUSSION

The work described in this study demonstrates that A. baumannii encodes a lipid A hydroxylase, LpxO, which mediates the 2-hydroxylation of the laurate transferred by LpxL_Ab._ This study demonstrates that LpxO-mediated 2-hydroxylation of lipid A protects the pathogen from CAMPs, mediates resistance to phagocytosis, and limits the activation of inflammatory responses by macrophages. Our findings also show that the *lpxO* mutant is attenuated in the G. mellonella infection model and that it induces a heightened antimicrobial response. Altogether, this evidence supports that LpxO is a *bona fide* immune evasion factor of A. baumannii.

Few Gram-negative bacteria are known to produce lipid A containing hydroxylated fatty acids, including pathogens such as *Salmonella, Klebsiella, Bordetella, Pseudomonas, Legionella,* and V. cholerae (14–17, 33–35), and also environmental bacteria such as *Marinomonas* and symbionts such as Vibrio fischeri (36–38). Mechanistically, lipid A hydroxylation is achieved either by the hydroxylation of fatty acids by the hydrolase catalyzed by LpxO (15, 16) or by transfer of a hydroxylated fatty acid by the acyl transferase LpxN (14). A. baumannii encodes an LpxO homolog which hydroxylates the laurate linked to the 2′ R-3-hydroxymyristoyl position of lipid A. Although our results and those of Boll et al. (7) conclusively demonstrate the function of A. baumannii LpxO, there is diversity on the lipid A position and fatty acid modified by other LpxO homologs. K. pneumoniae LpxO hydroxylates the myristate linked to the same position (16), whereas *Salmonella* LpxO hydroxylates the myristate linked to the 3′ R-3-hydroxymyristoyl position (15). The fact that lipid A hydroxylation seems to play a conserved role counteracting immune defenses independently of how it is achieved strongly suggests that incorporation of the hydroxyl group to lipid A is of the utmost importance here.

Our results clearly show that the 2-hydroxylation of lipid A is another of the mechanisms of A. baumannii to avoid the action of CAMPs and polymyxins. Of note, the hydroxylation of lipid A also mediates resistance to CAMPs in *Klebsiella* and V. cholerae (14, 16, 29), suggesting that this is an evolutionarily conserved mechanism of resistance against these antimicrobial agents. The fact that A. baumannii lipid A is constitutively hydroxylated supports the notion that LpxO-mediated lipid A modification can be considered part of the intrinsic resistome of this pathogen against these antimicrobials. Likewise, LpxM-dependent hepta-acylation of lipid A is also part of the A. baumannii intrinsic resistome (7). In contrast, the modification of A. baumannii lipid A with phosphoethanolamine and galactosamine is an inducible mechanism of resistance so far only found in clinical isolates as a result of colistin treatment (9–11). Future studies are warranted to assess the relative contribution of these mechanisms to counteract antimicrobial peptides and polymyxins. An extreme mechanism of inducible resistance is the loss of LOS due to mutations in lipid A biosynthetic genes (12). However, these strains increase the expression of other OM molecules, polysaccharides, and transporters, which may also play a role in the resistance to CAMPs (13, 39).

Another novel finding of this study is that LpxO-mediated lipid A hydroxylation attenuates the activation of defense responses. In G. mellonella, infection with the *lpxO* mutants triggered an upregulation of the expression of CAMPs, whereas in macrophages the *lpxO* mutant elicited a heightened inflammatory response. These results reinforce the notion that G. mellonella is a suitable surrogate infection model to assess the activation of early innate immune responses. The findings of this work are consistent with our earlier studies investigating K. pneumoniae LpxO-mediated lipid A modification (29, 30, 40), suggesting that the presence of a hydroxyl group on a lipid A secondary acyl chain is a conserved microbial immune evasion mechanism.

TLR4 signaling is essential for initiating inflammatory responses *in vitro* and *in vivo* to clear *Acinetobacter* infections (8, 41). Therefore, it is tempting to conclude that 2-hydroxylation of A. baumannii lipid A limits recognition by TLR4. However, our data showed that LpxO-mediated hydroxylation contributes to the expression of IL-10 in macrophages following infection. The fact that no differences were found in the immune responses activated by the wild type and *lpxO* mutant in *il10*^−/−^ macrophages strongly suggests that there are no differences in the recognition of LPS with or without hydroxylation. Collectively, this evidence is consistent with the notion that A. baumannii LpxO-mediated lipid A modification is an immune evasion mechanism dependent on the production of the anti-inflammatory cytokine IL-10. Providing additional support to this notion, the activation of the transcriptional factor CREB, necessary for the expression of IL-10 in macrophages (32), is significantly reduced in macrophages infected with the *lpxO* mutant. It is exciting to speculate that the induction of anti-inflammatory responses upon recognition of hydroxylated lipid A is an evolutionarily conserved mechanism to blunt immune responses. Future studies investigating other Gram-negative pathogens synthesizing hydroxylated lipid A are required to validate this hypothesis.

The World Health Organization has recently included A. baumannii in the list of pathogens for which new therapeutics are urgently needed. *Acinetobacter* infections are particularly troublesome within the health care setting and intensive care unit due to the organism's ability to resist disinfectants and to develop antibiotic resistance. The emergence of even pan-drug-resistant strains emphasizes the urgent need of developing alternative therapeutics. Among other possibilities and based on the results of this work, we put forward the idea that antivirulence therapeutics should be considered a viable option. Compounds directed to block LpxO may render A. baumannii susceptible to host CAMPs and may limit the attenuation of inflammatory responses. Interestingly, this approach might be useful to treat other infections caused by other multidrug-resistant pathogens producing hydroxylated lipid A, including *Pseudomonas* and *Klebsiella*.

## MATERIALS AND METHODS

### Bacterial strains and growth conditions.

All strains and plasmids used in this study are listed in Table 1. Strains were grown on lysogeny broth (LB) agar from frozen glycerol stocks at 37°C. Isolated colonies were used to inoculate LB broth at 37°C on an orbital shaker (180 rpm). When indicated, antibiotics were used at the following concentrations: carbenicillin (Carb), 50 μg/ml; kanamycin (Km), 25 μg/ml; chloramphenicol (Cm), 10 μg/ml; trimethoprim (Tp), 100 μg/ml; tetracycline (Tet), 12.5 μg/ml.

**TABLE 1:** Strains and plasmids used in this study

Bacterial strain or plasmid	Genotype or comment(s)	Source or reference
Strains		
A. baumannii		
ATCC 17978	Wild-type strain	American Type Culture Collection
ATCC 17978-Δ*lpxO*	ATCC 17978 with the *lpxO* gene inactivated	This study
ATCC 17978-Δ*lpxO*::Tn*7lpxO*	ATCC 17978, Δ*lpxO*; Tn7Km_17978*lpxO*Com integrated into *att*Tn*7* site, Km^r^	This study
E. coli		
C600	*thi thr leuB tonA lacY supE*	Laboratory collection
GT115	F*^−^ mcrA* Δ(*mrr-hsdRMS-mcrBC*) φ80*lacZ*ΔM15 Δ*lacX74 recA1 rpsL* (*strA*) *endA1 Δdcm uidA*(Δ*mluI*)::*pir-116 ΔsbcC-sbcD*	InvivoGen
β2163	F RP4-2-*Tc*::*Mu-d dapA*::(*erm-pir*), Km^r^, Em^r^	43
MG1655	F^−^ λ^−^ *ilvG rfb-50 rph-1*	Laboratory collection
BN1	W3110 Δ*eptA* Δ*lpxT* Δ*pagP*	19
BN1 Δ*lpxL*	BN1 Δ*lpxL*::FRT; the *lpxL* gene was inactivated	40
BN1 Δ*lpxL*::Tn*7lpxO*	BN1 Δ*lpxL*::FRT; Tn7Km_17978*lpxO*Com integrated into *att*Tn*7* site, Km^r^	This study
Plasmids		
pGEM-T Easy	Cloning plasmid, Amp^r^	Promega
pGPI-SceI-2	Suicide vector, R6Kgamma origin of replicaion, Mob^−^, carries an I-SceI endonuclease site, Tp^r^	42
pDAI-SceI-SacB	Expresses the I-SceI endonuclease gene, *sacB*, Tet^r^	42
pTNS2	T7 transposase expression vector, *oriR6K*, Amp^r^	49
pUC18R6kT-mini-Tn7TKm	pUC18R6KT-mini-Tn7TKm complementation vector, Amp^r^, Km^r^	44
pGEMΔ*lpxO*	pGEM-T Easy containing Δ*lpxO*; Amp^r^	This study
pGPI-SceiI-2Δ*lpxO*	pGPI-SceI-2 containing Δ*lpxO*, Tp^r^	This study
pUC18R6kT-mini-Tn7TKm-17978*lpxO*	pUC18R6kT-mini-Tn7TKm containing *lpxO* gene for complementation, Amp^r^, Km^r^	This study

To assess growth of A. baumannii strains, bacteria were grown as stated previously, and 5 μl of this culture was used to inoculate 250 μl of fresh LB. Absorbance readings of the optical density at 600 nm (OD_600_) were measured and recorded over a 24-h period with readings at 20-min intervals using a Bioscreen C automated microbial growth analyzer (MTX Lab Systems, Vienna, VA, USA). A total of 10 independent growth curves were obtained per strain.

### A. baumannii mutant construction.

Primers for mutant construction (see Table S1 in the supplemental material) were designed using the whole-genome sequence of A. baumannii ATCC 17978 (GenBank accession no. CP000521.1). The primer pairs *lpxO*_UP_FWD_, *lpxO*-UP_RVS_, *lpxO*_DOWN_FWD_, and *lpxO*_DOWN_RVS_ (Table S1) were used in two separate PCRs to amplify fragments flanking *A1S_0308* (A. baumannii *lpxO*) using *Ex Taq* polymerase (TaKaRa). Each amplicon had internal BamHI restriction sites added internally to the flanking regions. Gel-extracted *lpxO* UP and DOWN fragments were polymerized and amplified to generate a single PCR amplicon of the *lpxO* gene using primers *lpxO*_UP_FWD_ and *lpxO*_DOWN_RVS_. This 1,655-bp amplicon was cloned into pGEM-T Easy (Promega) to obtain pGEMΔ*lpxO* and transformed into E. coli C600 cells. After EcoRI digestion, the 1,655-bp amplicon was gel purified, cloned into EcoRI-digested Antarctic Phosphatase (New England Biolabs)-treated pGPI-SceI-2 suicide vector (42) to obtain pGPI-SceI-2Δ*lpxO*, and transformed into E. coli GT115 cells. pGPI-SceI-2Δ*lpxO* was transformed into E. coli β2163 (43), a diaminopimelate (DAP) auxotrophic donor strain with conjugative pili. pGPI-SceI-2Δ*lpxO* was mobilized into A. baumannii by conjugation. The cointegrant clones were selected using LB agar supplemented with Tp at 37°C. A second crossover event was performed by conjugating the pDAI-SceI-SacB plasmid (42) into an overnight culture grown to mid-exponential phase containing up to three Tp-resistant cointegrant clones. Exconjugants were selected with LB agar containing Tet at 37°C. Candidate clone colonies were screened for susceptibility to Tp on LB agar supplemented with Tp. Additionally, colony PCRs were performed using the *lpxO*_ UP_FWD_ and *lpxO*_DOWN_RVS_ primers. *sacB* allows curing of the pDAI-SceI-SacB vector by passaging the Tet-resistant, Tp-sensitive, PCR-confirmed colonies onto 6% sucrose LB agar without NaCl for 24 h at 30°C. The resulting colonies were screened for susceptibility to both Tet and Tp and confirmed with the aforementioned primers. This mutant was named the A. baumannii Δ*lpxO* mutant.

### Complementation of A. baumannii *lpxO* mutant.

To complement the A. baumannii Δ*lpxO* mutant, a DNA fragment comprising the coding and promoter regions of *lpxO* was amplified using Phusion high-fidelity DNA polymerase (New England Biolabs) and primers Ab_lpxO_UP_F1 and Ab_lpxO_DWN_R1 (Table S1). The amplicon was gel extracted and cloned into ScaI-digested (New England Biolabs), Antarctic Phosphatase-treated pUC18R6KT-mini-Tn7Km plasmid (44) to generate pUC18R6KT-mini-Tn7Km_17978*lpxO*Com. The resulting plasmid was then transformed into E. coli GT115 and subsequently into E. coli β2163. The transposase-containing pTNS2 and pUC18R6KT-mini-Tn7Km_17978*lpxO*Com were mobilized into the A. baumannii Δ*lpxO* mutant by triparental conjugation and, once recovered on LB DAP agar, were serially diluted on LB Km agar; the pTNS2-thermosensitive helper plasmid was cured by incubating for 6 h at 42°C, followed by overnight incubation at 37°C. Candidate colonies were screened for sensitivity to Tp. Correct integration of the Tn*7* transposon was confirmed by PCR using the primers Tn7-R and glmSF1. The presence of *lpxO* was confirmed by PCR using the primers *lpxO*_UP_FWD_ and *lpxO*_DOWN_RVS_. This strain was named the A. baumannii Δ*lpxO*::Tn*7lpxO* mutant.

pUC18R6KT-mini-Tn7Km_17978*lpxO*Com was also conjugated into E. coli BN1 and the BN1 Δ*lpxL* mutant as we have previously described (40).

### Lipid extraction and analysis.

Total lipids were extracted as described by Bligh and Dyer (45) and analyzed on silica gel 60 high-performance thin-layer chromatography (HPTLC) plates (Merck, Darmstadt, Germany). The plates were prewashed by solvent migration with a chloroform-methanol-acetic acid mixture (13:5:2) and dried thoroughly, samples were applied to the plate, and chromatography was performed in the same mixture of solvents. Plates were developed by charring with 15% sulfuric acid in ethanol at 180°C. Spots corresponding to lipids were quantified by densitometry using a GS-800 calibrated densitometer and Quantity One software (Bio-Rad).

### Fluorimetry.

The fluorescent probe *N*-phenyl-1-naphthylamine (NPN) (Sigma) has been used in outer membrane permeability studies. Fluorimetric assays were carried out as described by Martínez de Tejada et al. (46), with minor modifications. Exponentially growing bacteria were resuspended in 1 mM KCN–10 mM HEPES (pH 7.2) at an optical density at 600 nm of 0.1 and transferred to fluorimetric cuvettes. Fluorescence was monitored with an FLS920 Edinburgh fluorimeter with the following settings: excitation wavelength, 350 nm; emission wavelength, 420 nm; slit width, 5.0 nm. The results were expressed in relative fluorescence units.

### Lipid A extraction.

Lipid A was extracted using an isobutyric acid-ammonium hydroxide method (47) and analyzed with negative-ion MALDI-TOF mass spectrometry. Briefly, 10 ml of bacteria was grown to mid-exponential phase and washed three times with equal volumes of 10 mM phosphate buffer. The pellet was washed twice in 400 μl of chloroform-methanol (1:2 [vol/vol]) in a screw-cap test tube and centrifuged (2,000 × *g* for 15 min); the resulting pellet was resuspended in 400 μl of chloroform-methanol-water (3:2:0.25 [vol/vol/vol]) and centrifuged (2,000 × *g* for 15 min). The pellet was treated with 400 μl of isobutyric acid–1 M ammonium hydroxide (5:3 [vol/vol]) and incubated for 2 h at 100°C with occasional vortexing to hydrolyze the 3-deoxy-d-manno-octulosonic acid bond. The lipid A suspensions were then cooled in ice water and centrifuged (2,000 × *g* for 15 min), and the supernatant was transferred to a fresh screw-cap tube with an equal volume of deionized, sterile water and lyophilized. The lyophilized material was washed twice with 400 μl of absolute methanol and centrifuged (2,000 × *g* for 15 min). Lipid A was solubilized in 60 to 100 μl of chloroform-methanol-water (3:1.5:0.25 [vol/vol/vol]). A small volume of the solubilized lipid A was transferred to fresh 1.5-ml microcentrifuge tubes, desalted with a few grains of ion exchange resin (H^+^; Dowex 50W-X8), and briefly centrifuged. A 1-μl aliquot was deposited on a polished steel target plate for the dried droplet method. An equal volume of 2,5-dihydroxybenzoic acid (2,5-DHB) matrix (Bruker Daltonics, Inc.) was saturated in 100 mM citric acid (Sigma-Aldrich) or acetonitrile–0.1% trifluoroacetic acid (1:2 [vol/vol]) and allowed to air dry. Lipid A structural spectra were generated with a Bruker Autoflex Speed TOF/TOF mass spectrometer (Bruker Daltons Inc.) in negative reflectron mode with delayed extraction. All spectra were achieved with ion-accelerating voltage set at 20 kV, and resulting spectra were generated with an average of 300 shots. A peptide calibration standard (Bruker Daltonics, Inc.) was used to calibrate the MALDI-TOF mass spectrometer prior to analysis of each sample. Lyophilized E. coli MG1655 lipid A grown in LB broth at 37°C with identical extraction was used as an internal calibrant. Spectra are representative of at least three independent lipid A extractions.

### CAMP susceptibility assays.

To assess A. baumannii susceptibility to CAMPs, we performed a modified version of the sensitivity assay described by Llobet and coworkers (48). Each strain was grown to mid-exponential phase in LB broth, washed once with PBS, and diluted in liquid test medium composed of 1% tryptone soy broth (Oxoid), 10 mM phosphate buffer (pH 6.5), 100 mM sodium chloride (Sigma-Aldrich) to approximately 3 × 10^4^ CFU/ml. Aliquots (25 μl) of each strain were then mixed with 5 μl of either PBS or PBS containing antimicrobial peptides in 0.2-ml tubes. After 1 h of incubation at 37°C, 15 μl of the suspension was spread onto LB agar and incubated overnight at 37°C. Percent survival of the bacterial strains exposed to the antimicrobial peptides was calculated through comparison with the unexposed PBS controls. All assays were performed in duplicate on three independent experiments.

### Whole-blood phagocytosis assay.

Bacteria were grown at 37°C in 5 ml of LB broth on an orbital shaker (180 rpm) until mid-exponential phase and then harvested (3,000 × *g*, 15 min). Bacteria were washed once with sterile PBS and adjusted to an OD_600_ of 1.00. Three hundred microliters of fresh human blood (used within 30 min of collection with 10% citrate dextrose as an anticoagulant) was mixed with ∼1 × 10^7^ CFU/100 μl bacterial suspension and incubated on an orbital shaker at 37°C (180 rpm) for 3 h. Following incubation, serial dilutions were performed in sterile PBS and spread on LB agar. Bacterial counts recovered were then divided by the initial counts. Experiments were performed using blood from three individual donors, and for each blood sample the strains were tested in duplicate. Donors were all adult males, with no existing medical conditions or infections; none of the donors had used any anti-inflammatory medication for at least 24 h prior to blood withdrawal. Ethical approval for the use of blood from healthy volunteers to study bacterial killing was obtained from the Research Ethics Committee of the School of Medicine, Dentistry, and Biomedical Sciences (Queen’s Univeristy, Belfast, Ireland).

### Infection of G. mellonella larvae.

G. mellonella larvae were obtained from UK Waxworm Limited and stored at 12°C in the dark without dietary supplementation. All experiments were performed within 7 days of receipt, comprising randomly selected larvae with a healthy, nonmelanized appearance and within a weight range of 250 to 350 mg.

Infections were performed as described previously, with minor modifications (30). Briefly, bacteria were grown in 5 ml LB broth until mid-exponential phase (37°C, 180 rpm) and centrifuged (3,000 × *g*, 15 min), washed once with sterile PBS, and adjusted to 5 × 10^6^ CFU/ml. Larvae were surface disinfected with 70% (vol/vol) ethanol and then injected with 10 μl of the bacterial suspension, containing 5 × 10^4^ CFU, in the rear right proleg with a Hamilton syringe with a 27-gauge needle. A group of 10 larvae was also injected with 10 μl of sterile PBS to ensure death was not due to mechanical trauma. Larvae were placed in a 9.2-cm petri dish, kept at 37°C in the dark, and monitored every 24 h. *Galleria* organisms were considered dead when they were unresponsive to physical stimuli. Larvae were examined for pigmentation, and time of death was recorded. Pupa formation was observed among control larvae after 3 days, and experiments were ceased from this time point. The virulence assay was performed in triplicate (30 larvae total per strain).

### G. mellonella RNA extraction and RT-qPCR.

Larvae were infected with ∼ 5 × 10^4^ CFU of the three A. baumannii strains, incubated at 37°C for 12 h, and homogenized in 1 ml TRIzol reagent (Ambion) using a VDI 12 tissue homogenizer (VWR). Total RNA was extracted according to the manufacturer’s guidelines. Further purification was achieved by treating 1 μg of RNA with DNase I (Thermo Scientific) at 37°C for 30 min and then 65°C for 10 min in the presence of 50 mM EDTA. RNA was quantified using a Nanovue Plus spectrophotometer (GE Healthcare Life Sciences). cDNA was generated by retrotranscription of 1 μg of total RNA using Moloney murine leukemia virus (M-MLV) reverse transcriptase (Invitrogen) and random primers (Invitrogen). Ten nanograms of cDNA was used as the template in a 5-μl reaction mixture from a KAPA SYBR FAST quantitative PCR (qPCR) kit (Kapa Biosystems) and target primer mix (Table S1). Reverse transcription-qPCR (RT-qPCR) was performed using a Rotor-Gene Q (Qiagen) with the following thermocycling conditions: 95°C for 3 min for hot-start polymerase activation, followed by 40 cycles of 95°C for 5 s and 60°C for 20 s. Fluorescence of SYBR green dye was measured at 510 nm. Relative quantities of mRNAs were obtained using the comparative threshold cycle (ΔΔ*C_T_*) method by normalization to the amount of 18S rRNA.

### G. mellonella hemolymph microbroth assay.

Larvae were infected with ∼ 1 × 10^6^ heat-killed (65°C for 20 min) E. coli MG1655 organisms to prime the antimicrobial factors in the hemolymph and incubated at 37°C for 24 h. Hemolymphs from ten larvae were pooled in an ice-cold microcentrifuge tube containing 10 μl of 1 mg/ml *N*-phenylthiourea (Sigma). Five microliters of the hemolymph suspension was then used in the antimicrobial peptide assay previously described.

### Generation of iBMDM-derived macrophages.

To isolate BMDMs (bone marrow-derived macrophages), tibias and femurs from C57BL/6 or *IL-10*^−/−^ mice (C57BL/6 background) were removed using sterile techniques, and the bone marrow was flushed with fresh medium. To obtain macrophages, cells were plated in Dulbecco’s modified Eagle’s medium (DMEM) supplemented with 20% filtered L929 cell supernatant (a source of macrophage colony-stimulating factor) and maintained at 37°C in a humidified atmosphere of 5% CO_2_. Medium was replaced with fresh supplemental medium after 1 day. After 5 days, BMDMs were immortalized by exposing them for 24 h to the J2 CRE virus (carrying v-*myc* and v-Raf/v-Mil oncogenes, kindly donated by Avinash R. Shenoy, Imperial College London). This step was repeated 2 days later (day 7), followed by continuous culture in DMEM supplemented with 20% (vol/vol) filtered L929 cell supernatant for 4 to 6 weeks. The presence of a homogeneous population of macrophages was accessed by flow cytometry using antibodies for CD11b (clone M1/70; catalog number 17-0112-82; eBioscience) and CD11c (clone N418; catalog number 48-0114-82; eBioscience).

### Macrophage infections.

iBMDMs (cell line derived from wild-type NR9456 mice; BEI Resources, NIAID, NIH) were grown in DMEM (catalog number 41965; Gibco) supplemented with heat-inactivated fetal calf serum, 100 U/ml penicillin, and 0.1 mg/ml streptomycin (Gibco) at 37°C in a humidified 5% CO_2_ incubator. Cells were routinely tested for *Mycoplasma* contamination. For infections, iBMDMs were seeded to a density of 2.5 × 10^4^/well in 96-well plates, 5 × 10^5^/well in 12-well plates, and 1 × 10^6^/well in 6-well plates.

Bacteria were grown in 5 ml LB broth until mid-exponential phase at 37°C on an orbital shaker (180 rpm), recovered by centrifugation (3,000 × *g*, 15 min), and adjusted to an OD_600_ of 1.00 in sterile PBS corresponding to ∼1.2 × 10^8^ CFU/ml. Bacterial suspensions were serially diluted in PBS to count input of the bacteria and then UV irradiated at 15 J for 30 min, with confirmation of bacterial cell death by plating on LB agar. Infections were performed at a multiplicity of infection (MOI) of 20 bacteria per iBMDM. Synchronization of the infection was performed by centrifugation (200 × *g* for 5 min). All infections were performed in duplicate and repeated three independent times.

### Macrophage RNA isolation and RT-qPCR.

Infections were performed in 6-well plates with UV-killed A. baumannii for 5 h. Cells were washed three times with prewarmed sterile PBS, and total RNA was extracted from the cells in 1 ml of TRIzol reagent (Ambion) according to the manufacturer’s instructions. Extracted RNA was treated with DNase I (Roche) and precipitated with sodium acetate (Ambion) and ethanol. RNA was quantified using a Nanovue Plus spectrophotometer (GE Healthcare Life Sciences).

cDNA was generated by retrotranscription of 1 μg of total RNA using M-MLV reverse transcriptase (Invitrogen) and random primers (Invitrogen). Ten nanograms of cDNA was used as a template in a 5-μl reaction mixture from a KAPA SYBR FAST qPCR kit (Kapa Biosystems) and target primer mix (Table S1). RT-qPCR was performed using a Rotor-Gene Q (Qiagen) with the following thermocycling conditions: 95°C for 3 min for hot-start polymerase activation, followed by 40 cycles of 95°C for 5 s and 60°C for 20 s. Fluorescence of SYBR green dye was measured at 510 nm. Relative quantities of mRNAs were obtained using the ΔΔ*C_T_* method by using hypoxanthine phosphoribosyltransferase 1 (*hprt*) gene normalization.

### Transfection conditions.

For transfection of siRNAs, ∼1 × 10^6^ iBMDMs (6-well format) were transfected in suspension with 20 nM siRNA using Lipofectamine and Opti-MEM I (ThermoFisher) in a final volume of 2 ml. AllStars negative-control siRNA (Qiagen) or ON-TARGETplus SMARTpool siRNA targeting CREB1 (no. L-040959-01; Dharmacon) was used to transfect cells for 16 h. Cells then were mock infected with PBS or infected with UV-killed A. baumannii ATCC 17978 as described previously. RNA was collected and RT-qPCR analyses were performed as previously described. Knockdown efficiency (Fig. S4) was determined using primers for *creb* (Table S1).

### Immunoblots.

Proteins were resolved on standard 10% SDS-PAGE gels and electroblotted in a semidry manner onto nitrocellulose membranes. Membranes were subsequently blocked with 3% (wt/vol) bovine serum albumin in TBS-Tween (TBST), and specific antibodies were used to detect protein using chemiluminescence reagents and a G:BOX Chemi XRQ chemiluminescence imager (Syngene).

All antibodies were probed with a secondary goat anti-rabbit/mouse antibody conjugated to IgG horseradish peroxidase, diluted 1:5,000 (no. 170-6515/170-6516; Bio-Rad). Antibodies used in this study were anti-IκBα (anti-rabbit, 1:1,000; no. 4812S; Cell Signaling), anti-phospho-JNK (anti-rabbit, 1:1,000; no. 9251S; Cell Signaling), anti-phospho-ERK (anti-rabbit, 1:1,000; no. 9101; Cell Signaling), anti-phospho-CREB (anti-rabbit, 1:1,000; no. sc-7978-R; Santa Cruz Biotechnology), and anti-phospho-p38 (anti-rabbit, 1:1,000; no. 4511; Cell Signaling). To ensure that equal amounts of proteins were loaded, blots were reprobed with α-tubulin (anti-mouse, 1:4,000; no. T5168; Sigma-Aldrich).

To detect multiple proteins, membranes were reprobed after stripping of previously used antibodies using a pH 2.2 glycine‐HCl–SDS buffer.

Bands were analyzed using ImageJ with the Histogram analysis tool, and results were expressed as a percentage of the intensity of the band found in the samples from cells infected with the wild-type strain.

### Quantification of cytokines.

Infections were performed in 12-well plates (5 × 10^5^ cells per well) at an MOI of 20:1 UV-killed bacteria. After 5 h of infection, supernatants were collected and spun down at 12,000 × *g* for 5 min to remove any debris. TNF-α in the supernatants was determined using a murine TNF-α standard TMB enzyme-linked immunosorbent assay development kit (no. 900-T54; PeproTech) according to the manufacturer’s instructions. All experiments were performed in duplicate, and three independent experiments were conducted.

### Statistical analysis.

Statistical analyses were performed using one-way analysis of variance (ANOVA) with Bonferroni corrections, the one-tailed *t* test, or, when the requirements were not met, the Mann-Whitney U test. *P* values of <0.05 were considered statistically significant. Normality and equal variance assumptions were tested with the Kolmogorov-Smirnov test and the Brown-Forsythe test, respectively. Survival analyses were undertaken using the log‐rank (Mantel-Cox) test with Bonferroni correction for multiple comparisons (α = 0.008). All analyses were performed using GraphPad Prism for Windows (version 6.01) software.

## Supplementary Material

Supplemental file 1

Supplemental file 2
