# Supplementary material for: 2-Hydroxylation of Acinetobacter baumannii Lipid A Contributes to Virulence
Source: Infect Immun. 2019 Mar 25;87(4):e00066-19. doi: 10.1128/IAI.00066-19 (PMC6434125; doi:10.1128/IAI.00066-19)
Supplement: Supplemental file 1 [file IAI.00066-19-s0001.pdf]

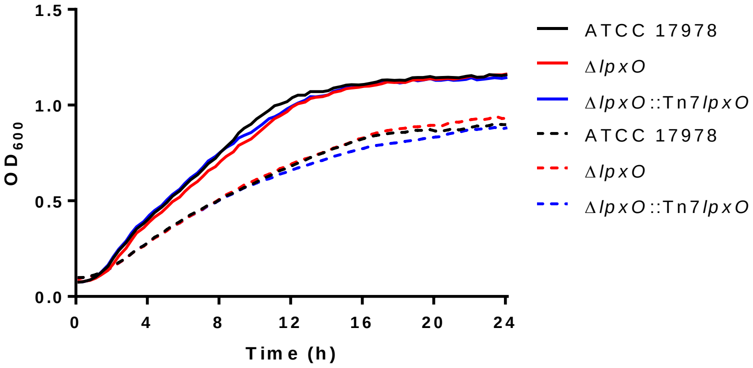


**Supplementary figure 1. Growth kinetics of *A. baumannii* strains.**

Growth kinetics of *A. baumannii* ATCC 17978 (ATCC 17978, black), *A. baumannii* Δ*lpxO* (Δ*lpxO*, red, *A. baumannii* ΔlpxO::Tn7*lpxO* (Δ*lpxO*::Tn7*lpxO, blue*) cultured in LB broth (LB, solid lines)) and M9 minimal medium (dotted lines) over 24 hours at $37^{0}$C. Values are presented as the mean ± SD of five independent experiments.

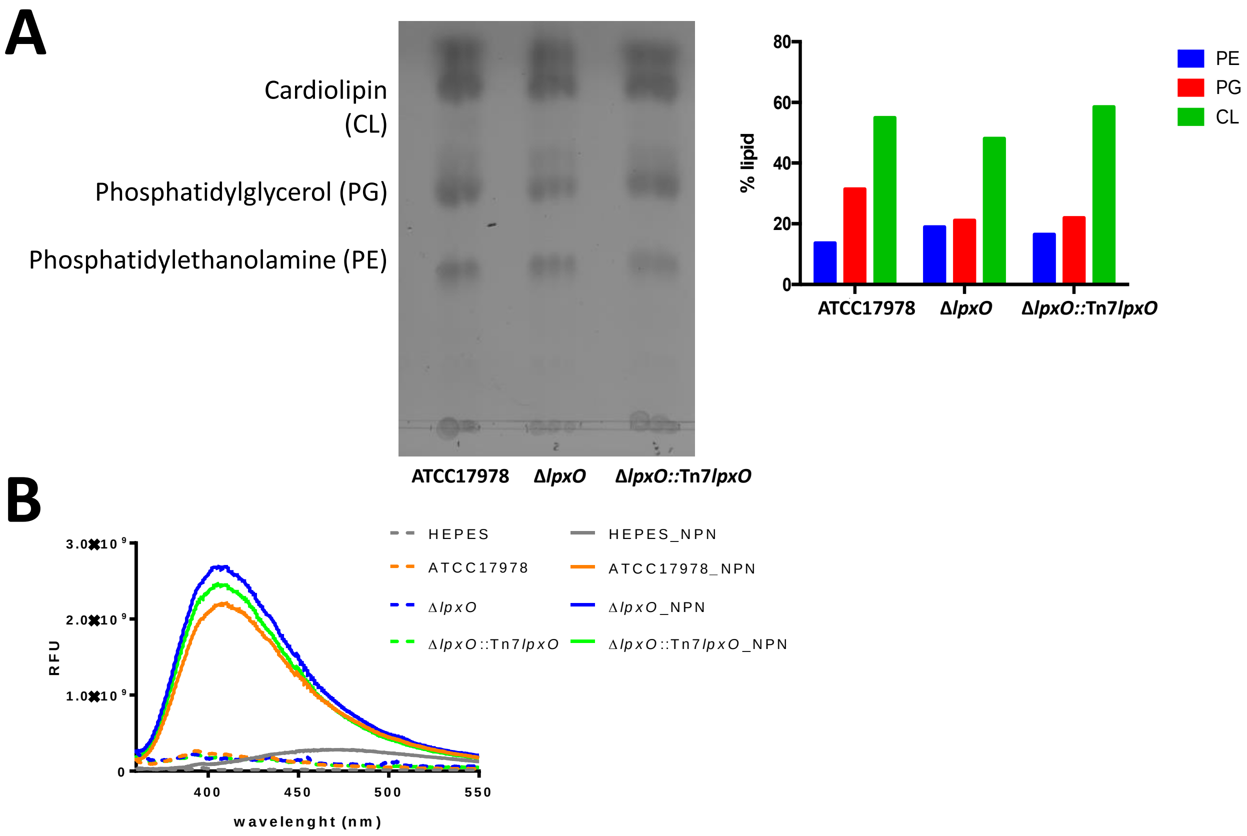


**Supplementary figure 2**. ***lpxO* deletion in *A. baumannii* does not result in outer membrane alterations.**

(A) Analysis by thin layer chromatography of *A. baumannii* cell envelope phospholipids; right panel shows the percent of a given phospholipid per total lipid content. (B) Partition of the NPN probe in the outer membrane of *A. baumannii* ATCC 17978 (ATCC 17978), *A. baumannii* Δ*lpxO* (Δ*lpxO*), (D) *A. baumannii* ΔlpxO::Tn7*lpxO* (Δ*lpxO*::Tn7*lpxO*). Results are representative of three independent experiments.

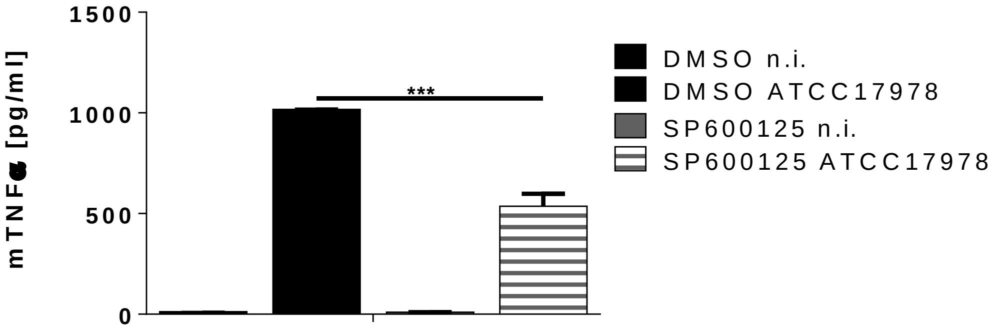


**Supplementary figure 3**. **JNK controls inflammatory response in *A. baumannii* infected macrophages.**

TNFα secretion upon infection in iBMDM macrophages was analysed in the presence of the MAPK JNK inhibitor (SP600125, 10 µM, 2 hours before infection or DMSO [vehicle solution]). Macrophages were stimulated for 5 hours with UV-killed *A. baumannii* ATCC17978 and TNFα levels were determined in the supernatant of infected cells. ***, $P<0.001$; for the indicated comparison using the two-way ANOVA with Bonferroni contrasts.

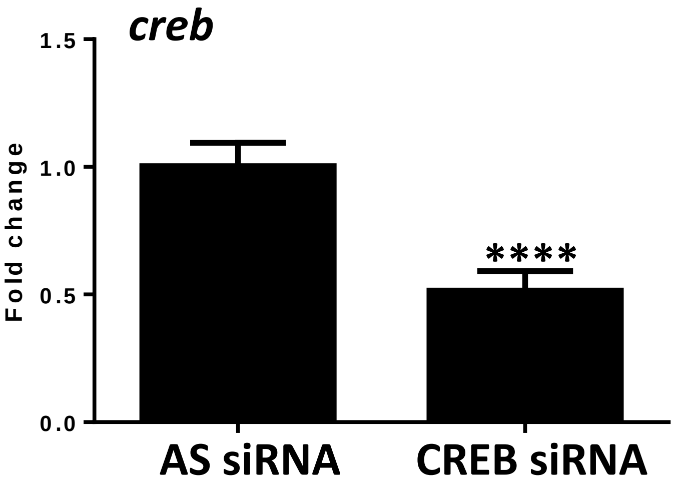


**Supplementary figure 4**. Knockdown efficiency of CREB by siRNA.

Transcript levels of *creb* relative to AllStars control siRNA-transfected cells as determined by RT-qPCR after gene normalization. Values are presented as the mean ± SD of three independent experiments measured in duplicate. ****$P < 0.0001$ one-tailed *t* test.
