# Supplementary material for: 2-Hydroxylation of Acinetobacter baumannii Lipid A Contributes to Virulence
Source: Infect Immun. 2019 Mar 25;87(4):e00066-19. doi: 10.1128/IAI.00066-19 (PMC6434125; doi:10.1128/IAI.00066-19)
Supplement: Supplemental file 2 [file IAI.00066-19-s0002.pdf]

Table S1. Primers used in this study.

Method and target gene	Primer	Sequence (5'–3')
Mutagenesis and complementations		
A. baumannii		
lpxO	Ab_lpxO_UP_F1	GTA CAG CTC CGG TAC ACA TTT A
	Ab_lpxO_DWN_F1(BamHI)	[1] GGA TCC TGG TTC CTC ATG CTT GGA AT
	Ab_lpxO_UP_R1(BamHI)	[1] GGA TCC TAG GGT TGA TTC GGT ACC TTT G
	Ab_lpxO_DWN_R1	ATA GGA AAC TAG CCC GAC AAA G
	KpnLpxL1ComF	GCC GGT TAA AAA GCA GCC AA
	KpnLpxL1ComR	AGG AAA TAA ACG GCG GGC AT
Tn7 insertion	Ab_glmS_F1	TTT GCT GAT GAA AAT AGC GG
	Tn7_Right	CAC AGC ATA ACT GGA CTG ATT TC
E. coli		
Tn7 insertion	E.coli_glmS_Up	TCG ACT GGG CGT ACA AAA CC
	E.coli_glmS_Down	CGG GAA ACC ATA CCG GAG TT
	EcoliLpxL_check_ F	GCA CAC TAA TTA TGC GCC CG
	E.coli_pTN7L_F1	ATT AGC TTA CGA CGC TAC ACC C
	E.coli_pTN7R_R1	CAC AGC ATA ACT GGA CTG ATT TC
qPCR		
hprt	mHPRT-F1	GAT CAG TCA ACG GGG GAC AT
	mHPRT-R1	GGT CCT TTT CAC CAG CAA GC
tnfα	mTNF-α-F1	TTC TGT CTA CTG AAC TTC GGG GTG ATC GGT CC
	mTNF-α-R1	GTA TGA GAT AGC AAA TCG GCT GAC GGT GTG GG
Il10	mIL-10_F1	GGA CTT TAA GGG TTA CTT GGG TTG CC
	mIL-10_R1	CAT GTA TGC TTC TAT GCA GTT GAT GA
creb	mCREB1_F1	TAA CAG TGC CAA CCC CCA TTT
	mCREB1_R1	CCT GTA CCC CAT CCG TAC CA
18S	Galleria_18S_F1	ATG GTT GCA AAG CTG AAA CT
	Galleria_18S_R1	TCC CGT GTT GAG TCA AAT TA
lysozyme	Gall_Lysozyme_F1	TCC CAA CTC TTG ACC GAC GA
	Gall_Lysozyme_R1	AGT GGT TGC GCC ATC CAT AC
galiomycin	Galiomycin_F1	CCT CTG ATT GCA ATG CTG AGT G
	Galiomycin_R1	GCT GCC AAG TTA GTC AAC AGG
gallerimycin	Gallerimycin_F1	CGC AAT ATC ATT GGC CTT CT
	Gallerimycin_R1	CCT GCA GTT AGC AAT GCA CTC

[1] BamHI site underlined.
